# Cardiotoxicity Conundrum: Distinguishing Immune Checkpoint Inhibitor Myocarditis From Fluorouracil Cardiotoxicity in Concurrent Therapy

**DOI:** 10.1016/j.jaccas.2025.104002

**Published:** 2025-07-09

**Authors:** Vivian Hu, Ishan Naidu, Nausheen Singh, Oludamilola Akinmolayemi, Gagan Sahni

**Affiliations:** a Department of Medicine, Icahn School of Medicine at Mount Sinai, New York, New York, USA; b Mount Sinai Fuster Heart Hospital, Icahn School of Medicine at Mount Sinai, New York, New York, USA

**Keywords:** cardio-oncology, cardiotoxicity, fluorouracil, immune checkpoint inhibitor, myocarditis

## Abstract

**Background:**

Immune checkpoint inhibitors (ICIs) have transformed cancer treatment over the past decade; however, it is crucial to recognize their rare but clinically significant cardiotoxicities. These agents are often combined with other chemotherapeutics carrying distinct cardiotoxic profiles.

**Case Summary:**

A 37-year-old man presented with cardiogenic shock shortly after initiating ICI and fluorouracil (5FU) therapy. He was treated concurrently for suspected ICI myocarditis and 5FU cardiotoxicity with rapid improvement. Given higher suspicion for 5FU as the culprit, he was rechallenged successfully with nivolumab.

**Discussion:**

Distinguishing between overlapping cardiotoxicities and establishing causality can be challenging in patients receiving combination therapy. Diagnostic tests alone may be insufficient to confirm a diagnosis and should be complemented by a thorough assessment of risk factors and clinical presentation.

**Take-Home Message:**

This case highlights the importance of prompt and accurate identification of a causative agent for cardiotoxicity with consequential impact on future therapeutics and cancer prognosis.

## Graphical Abstract

**Figure:**
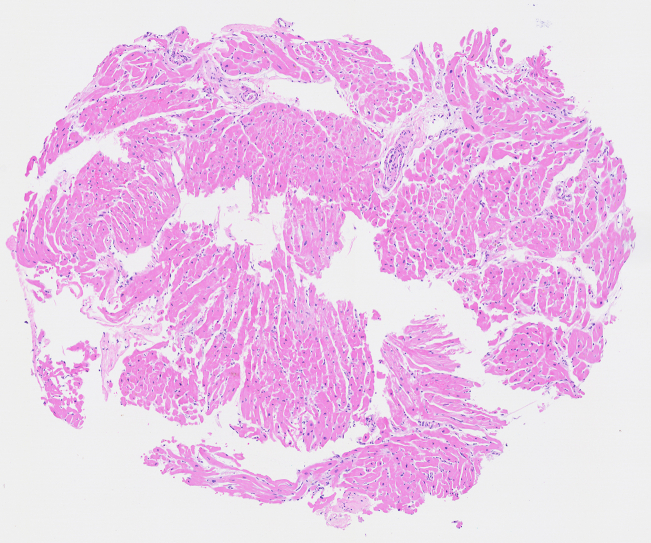

## History of Presentation

A 37-year-old man with metastatic gastroesophageal junction adenocarcinoma to the liver presented with nausea, chest pain, and dyspnea 48 hours after initiating cycle 1 of leucovorin, fluorouracil, oxaliplatin (FOLFOX) and nivolumab. He received 750 mg leucovorin, 160 mg oxaliplatin, 240 mg nivolumab, and 750 mg fluorouracil (5FU) followed by a continuous infusion totaling 4,600 mg.

**Take-Home Messages**

• Timely identification of potential cardiotoxicity in combination cancer therapies is essential to prevent severe complications.
• Comprehensive assessment of clinical presentation, risk factors, and diagnostic testing is critical to accurately determine a causative agent and guide treatment decisions.
• An interdisciplinary approach is necessary if considering rechallenge to ensure patient safety.

His examination was notable for tachycardia, hypotension, hypoxia, and bilateral rales without oral mucositis. Laboratory findings included elevated high-sensitivity troponin I (634 ng/L; reference: <35 ng/L), B-type natriuretic protein (BNP 700.8 pg/mL; reference: <100 pg/mL), white blood cell count (12,400/μL; reference: 4,500-11,000/μL), and lactate (2.6 mmol/L; reference: 0.5-2.2 mmol/L), with normal creatine phosphokinase, erythrocyte sedimentation rate, C-reactive protein, and liver function tests. Electrocardiography without prior baseline showed sinus tachycardia at 130 beats/min with isolated T-wave inversion in V_2_ and up-sloping ST-segment depressions in V_3_ and V_4_ without reciprocal ST-segment elevations. Point-of-care ultrasound showed globally reduced left ventricular systolic function with an approximate ejection fraction of 30%. Computed tomography angiography (CTA) of the chest ruled out pulmonary embolism (PE). He was admitted to the cardiac intensive care unit for cardiogenic shock.

## Past Medical History

The patient was diagnosed with metastatic gastroesophageal junction adenocarcinoma 1 month earlier. His family history was significant for a myocardial infarction in his father in his 40s.

## Differential Diagnosis

Differential diagnosis included 5FU cardiotoxicity, ICI myocarditis, 5FU-associated takotsubo cardiomyopathy (TCM), idiosyncratic reaction to 5FU, acute coronary syndrome (ACS) in the setting of thrombotic occlusion or vasospasm due to 5FU, and PE.

## Investigations

**Figure 1:**
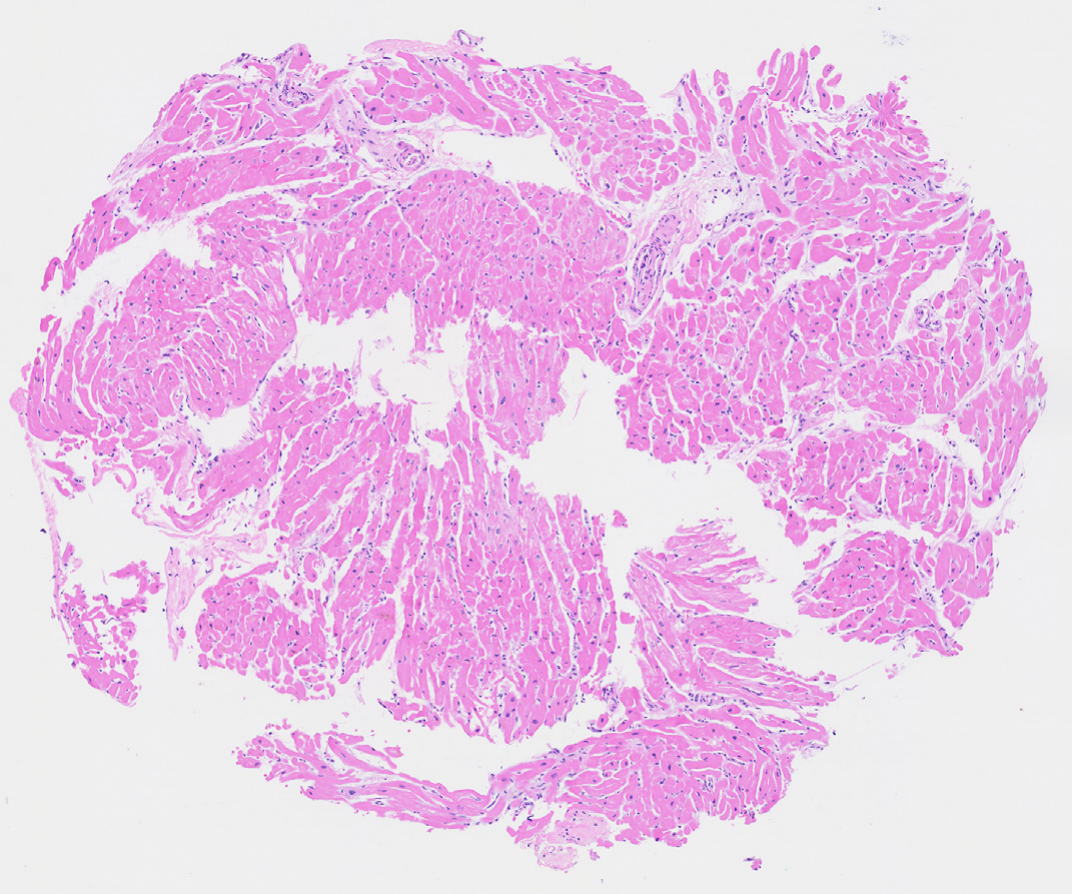
Endomyocardial Biopsy of the Right Ventricle Myocardial tissue with minimal hypertrophic changes and without inflammatory cell infiltrates or granulomas.

ACS was less likely in this young patient with low risk of arteriosclerosis cardiovascular disease, and PE was ruled out with the use of CTA. His presentation shortly after FOLFOX and nivolumab initiation raised suspicion for medication-related toxicity. He was planned for simultaneous treatment of suspected ICI myocarditis with 1 g methylprednisolone daily for 3 days and of 5FU cardiotoxicity with uridine triacetate for 20 doses. He received the first dose of methylprednisolone quickly. Right heart catheterization on 2.5 μg/kg/min dobutamine, 1 hour after his first methylprednisolone dose and before uridine triacetate revealed normal cardiac hemodynamics (mean right atrial pressure 2 mm Hg, mean pulmonary artery pressure 16 mm Hg, mean pulmonary capillary wedge pressure 12 mm Hg, mixed venous saturation 75%, cardiac output by Fick 6 L/min, cardiac index by Fick 3.3 L/min/m^2^). Endomyocardial biopsy did not show inflammatory cell infiltrates or granulomas (Figure 1). Transthoracic echocardiography (TTE) on dobutamine, 4 hours after his first methylprednisolone dose and before uridine triacetate, demonstrated improving cardiac function with diffusely reduced left ventricular ejection fraction (LVEF) of 35% to 40%. There was no evidence of atrial ballooning, midventricular ballooning, basal hyperkinesis, or right ventricular involvement. Cardiac magnetic resonance imaging on day 4 off dobutamine, after 3 doses of methylprednisolone and 7 doses of uridine triacetate, showed normal biventricular function, with an LVEF of 54%, no myocardial scarring or necrosis on late gadolinium enhancement imaging, no myocardial edema on T2-weighted imaging, and no pericardial thickening, but elevated T1 time (1,144 ms) and T2 time (54 ms), more pronounced in the septal segments, and trivial pericardial effusion. His workup further revealed normal dihydropyrimidine dehydrogenase (DPD) activity.

## Management

**Figure 2:**
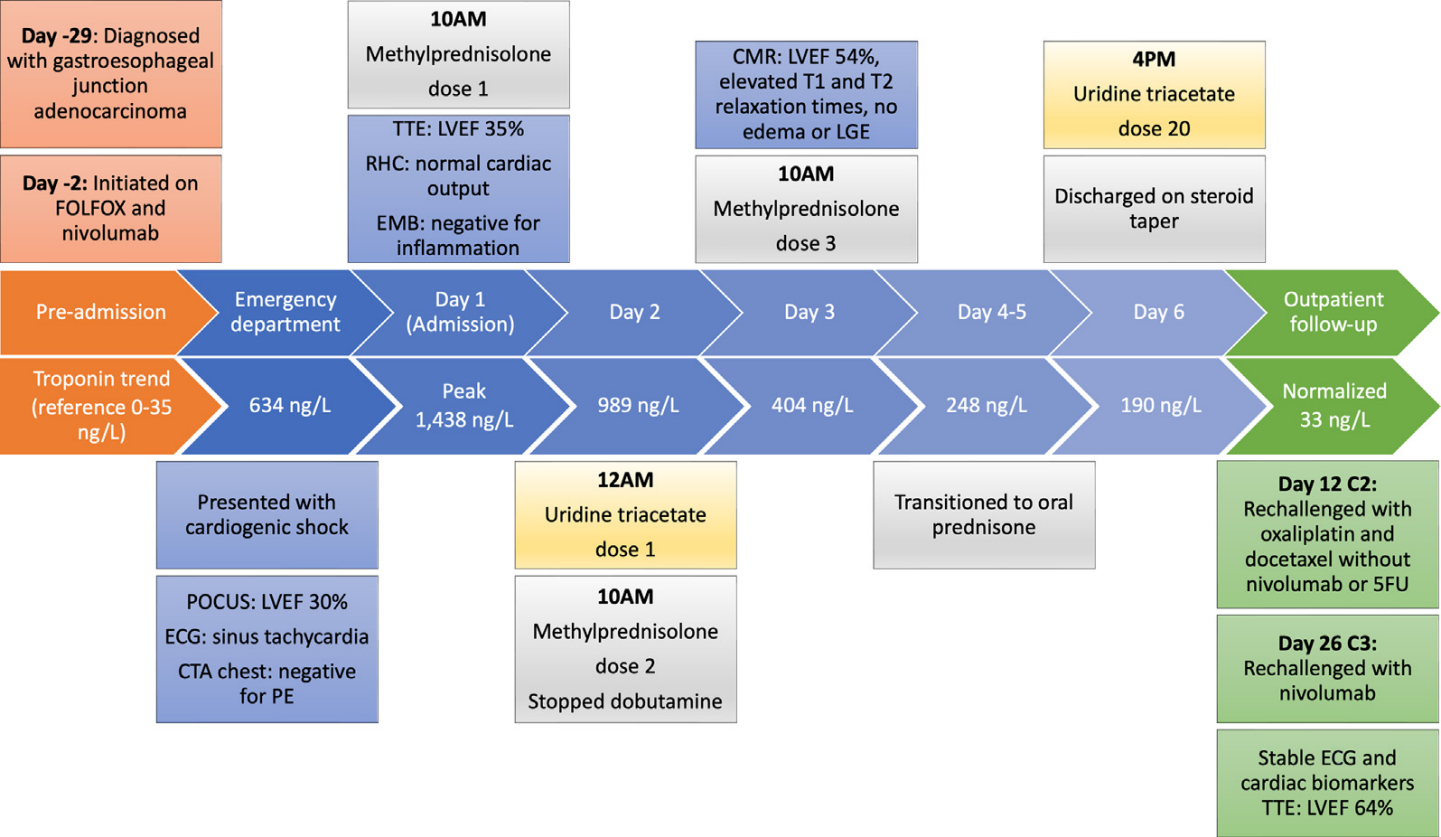
Summary of Events From Preadmission to Outpatient Follow-Up The course of treatment, diagnostic testing, and cardiac biomarkers trend. 5FU = fluorouracil; CMR = cardiac magnetic resonance; CTA = computed tomographic angiography; ECG = electrocardiography; EMB = endomyocardial biopsy; FOLFOX = leucovorin, fluorouracil, oxaliplatin; LVEF = left ventricular ejection fraction; POCUS = point of care ultrasound; RHC = right heart catheterization; TTE = transthoracic echocardiography.

Before receiving uridine triacetate, he had symptomatic improvement and down-trending cardiac biomarkers (Figure 2). His tachycardia resolved without evidence of ventricular arrhythmia or conduction disease. He was weaned from dobutamine 24 hours after the first methylprednisolone dose and 10 hours after the first uridine triacetate dose. He was changed to 1 mg/kg oral prednisone daily after 3 doses of pulse steroids, completed 20 doses of uridine triacetate, and was discharged on a rapid prednisone taper.

## Outcome and Follow-Up

At his follow-up appointment 11 days after his initial presentation, he reported improved exercise tolerance without reoccurrence of symptoms. His cardiac biomarkers normalized, and he was initiated on 25 mg losartan daily. After multidisciplinary discussions among his cardio-oncologist, medical oncologist, and oncology-pharmacist, he was rechallenged for cycle 2 with 130 mg oxaliplatin and 75 mg docetaxel. His electrocardiographic and cardiac biomarkers remained stable. Repeated TTE demonstrated normal biventricular function with an LVEF of 64% and normal global longitudinal strain. Nivolumab (240 mg) was added for cycle 3 without further adverse cardiac events.

## Discussion

Identifying a causative agent is challenging in a patient receiving multiple potentially cardiotoxic cancer therapies. ICI myocarditis is relatively rare, with a prevalence of 1.14% and median time of onset of 34 days.^1^ Mahmood et al found that 94% of ICI myocarditis patients had elevated troponin and 66% elevated BNP. Increased admission, peak, and discharge troponins were associated with higher risk of major adverse cardiac events, supporting the prognostic value of trending troponin through the disease course.^1^

The reported incidence of 5FU cardiotoxicity varies widely from 1% to 35% and develops early, with more than 70% presenting during the first cycle.^2^ The more common side-effect of 5FU is gastrointestinal toxicity, which can accompany cardiac manifestations.^3^ Cardiac biomarkers are less sensitive, because troponin elevation may be identified in early toxicity during 5FU infusion and may become undetectable in late-stage toxicity, 1 to 2 days after infusion.^4^ Although ICI myocarditis carries a high mortality risk, life-threatening cardiotoxicity from 5FU, including cardiogenic shock, ACS, and malignant arrhythmias, are less common.^1^^,^^3^ A retrospective study found no significant difference in overall survival in patients who developed 5FU cardiotoxicity compared with those who did not.^2^

The present patient’s initial presentation did not clearly identify a causative agent. The rapid improvement in troponin level and early onset with nausea may have represented gastrointestinal toxicity associated with 5FU, whereas the cardiogenic shock was more suggestive of ICI myocarditis. Improvement in cardiac biomarkers before receiving uridine triacetate raised suspicion for ICI myocarditis; however, it is difficult to distinguish whether this recovery was due to discontinuation of the offending agent or initiation of steroid treatment.

A broader spectrum of 5FU-related effects should be considered in addition to the more classically described cardiotoxicity and vasospasm. The patient’s chest pain, elevated cardiac enzymes, cardiomyopathy, and rapid recovery raise concern for 5FU-associated TCM although TTE did not show classic findings. A single-center retrospective study suggests that stress cardiomyopathy in cancer patients is multifactorial, driven by the combined effects of chemotherapy, surgical intervention, and emotional stress. The median time to resume cancer treatment was 20 days, and no patients experienced recurrence of stress cardiomyopathy, potentially supporting the safety of 5FU rechallenge after TCM.^5^ Our patient’s down-trending biomarkers after steroids also raise concern for an adverse immunologic response as in ICI myocarditis or idiosyncratic reactions to 5FU, particularly when used in combination with oxaliplatin.^1^^,^^6^

Risk factors for ICI myocarditis and 5FU cardiotoxicity are not well defined. One study found combination ICI therapy and diabetes to be more common in ICI myocarditis cases.^1^ The risk of 5FU cardiotoxicity is increased in multiagent chemotherapy, concurrent radiation therapy, and protracted infusion.^3^ Another study found that patients diagnosed with 5FU cardiotoxicity were an average 10 years younger and less likely to have baseline cardiovascular risk factors or ischemic disease.^2^ The most studied genetic risk factor for 5FU cardiotoxicity is DPD enzyme deficiency. This enzyme catalyzes the rate-limiting step of 5FU metabolism, and its deficiency or absence predisposes patients to severe 5FU toxicity.^7^

Coronary CTA and coronary artery calcium scoring are useful imaging modalities to rule out coronary artery disease in common cancer-associated ACS mimickers, such as TCM, 5FU-associated vasospasm, and ICI myocarditis. The negative predictive value for atherosclerotic cardiovascular disease with the use of coronary CTA is helpful for risk stratification even in younger patients with low suspicion for ACS.^8^ Although suboptimal for evaluation of coronary artery disease, a CTA of the chest obtained on admission in our patient did not show any overt atherosclerosis.

Management of ICI myocarditis and 5FU toxicity starts with cessation of the medication. The mainstay of treatment for ICI myocarditis is immunosuppression with high-dose steroids.^1^ Uridine triacetate is authorized for urgent treatment of 5FU overdose and should be initiated within 96 hours of infusion completion, but its high cost and limited availability can be a barrier to timely treatment.^7^ There is a paucity of data specific to guideline-directed medical therapy use in cancer therapy–related cardiomyopathy; therefore, current management aligns with that of heart failure with reduced ejection fraction.^9^

The decision to rechallenge with an ICI was complex, requiring multidisciplinary discussion and careful risk assessment owing to the high mortality associated with ICI myocarditis. Although the endomyocardial biopsy was negative and the patient met only minor criteria according to the IC-OS 2021 consensus, ICI myocarditis could not be definitively excluded.^10^ DPD testing provides an additional data point: Although a deficiency lends support to a diagnosis of 5FU toxicity, a normal result does not eliminate risk. In the absence of a single definitive diagnostic test, our patient’s overall clinical presentation and investigations favored a diagnosis of 5FU cardiotoxicity.

## Conclusions

This case highlights the importance of early recognition of potential cardiotoxicity in combination cancer therapies and the challenges of identifying a causative agent when multiple drugs with distinct mechanisms of toxicity are involved.

## Funding Support and Author Disclosures

The authors have reported that they have no relationships relevant to the contents of this paper to disclose.
